# Prognostic and Immune-Infiltrate Significance of miR-222-3p and Its Target Genes in Thyroid Cancer

**DOI:** 10.3389/fgene.2021.710412

**Published:** 2021-10-19

**Authors:** Taofeng Zhang, Yihuan Chen, Weixun Lin, Jiehua Zheng, Yiyuan Liu, Juan Zou, Jiehui Cai, Yaokun Chen, Zhiyang Li, Yexi Chen

**Affiliations:** ^1^ Department of General Surgery, The Second Affiliated Hospital of Shantou University Medical College, Shantou, China; ^2^ Department of Ultrasound in Obstetrics and Gynecology, The Second Affiliated Hospital of Shantou University Medical College, Shantou, China; ^3^ Department of Breast Disease Research Center, Medical Research Institute of Shantou Doctoral Association, Shantou, China

**Keywords:** miR-222-3p, thyroid cancer, prognostic significance, immune infiltration, target gene, diagnostic significance

## Abstract

Thyroid cancer (THCA) is a common endocrine malignancy. With increasing incidence and low mortality, balancing the therapeutic approach is an inevitable issue. This study aimed to confirm the role of miR-222-3p and its target genes in THCA survival and immune infiltration. From different expression analyses based on the GEO and TCGA databases, we predicted and subsequently identified the key target genes of miR-222-3p. We then explored the expression, enrichment, pairwise correlation, protein expression, survival analysis, principal component analysis, and immune significance of the critical genes using bioinformatics analysis. The present study demonstrated that *NEGR1*, *NTNG1*, *XPNPEP2*, *NTNG2*, *CD109*, *OPCML*, and *PRND* are critical genes. The miR-222-3p was highly expressed, probably leading to low *NEGR1* and high *PRND* expression in THCA tissues. Low *NEGR1* expression indicated favorable prognosis in THCA patients, and high *PRND* expression indicated poor prognosis. Seven critical genes were significantly related to gender, age, race, tumor stage, and lymph node metastasis. In addition, the seven-gene biomarker exhibited a certain diagnostic value. Finally, *CD109* expression was closely correlated with immune cells, especially B cells and CD4^+^ T cells. The miR-222-3p and its critical target genes could be promising biomarkers for the prognosis of THCA and may emerge as key regulators of immune infiltration in THCA.

## Introduction

Thyroid cancer (THCA) is one of the most common endocrine malignancies and is a complex disease resulting from radiation exposure, abnormal hormone levels, abnormal iodine uptake, and genetic susceptibility(Cabanillas et al., 2016). Globally, among all types of cancers, the incidence of THCA ranks fifth worldwide, mostly due to the popularization of diagnostic imaging and surveillance(Cabanillas et al., 2016; Bray et al., 2018). Even though the prognosis of THCA is encouraging, avoiding overtreatment of low-risk patients and undertaking aggressive treatment for high-risk patients to improve prognosis can be challenging(Cabanillas et al., 2016). Indolent THCAs have low mortality, whereas other types may be aggressive, for instance anaplastic thyroid cancer. Therefore, the identification of new biomarkers for predicting the prognosis of THCA effectively is crucial for individual treatment.

MicroRNAs (miRNAs) are a class of endogenous non-coding RNA molecules, 19–25 nucleotides in length. Mature microRNAs can target the exact complementary sequence of the 3′-untranslated region (3′-UTR), resulting in the inhibition of translation and degradation of messenger ribonucleic acid (mRNA) (Bartel, 2004; Saliminejad et al., 2019). Many miRNAs are important regulators in multiple cancers, including THCA, can distinguish benign from malignant nodules(Di Leva et al., 2014; Dai et al., 2020). In recent years, studies have shown that miR-222 is highly expressed in THCA and is probably related to the occurrence, development, and prognosis(Song et al., 2017) (Lima et al., 2017; Zhang et al., 2017; Gomez-Perez et al., 2019). For example, Huang et al. found that miR-222 promotes tumor invasion and metastasis in papillary thyroid cancer by targeting *PPP2R2A*(Huang et al., 2018). However, as a familiar member of miRNA in THCA, there are few studies that have focused on the target genes of miR-222-3p, and the clinical and prognostic value of the target genes in THCA requires further study.

This study investigated the differential miR-222-3p expression in THCA tissues and non-cancerous thyroid tissues, acquired the differentially expressed genes (DEGs) in the THCA dataset of the Cancer Genome Atlas (TCGA), predicted the possible target genes of miR-222-3p using miRWalk3.0, and identified the common genes of DEGs and the possible target genes as the consensus genes. Subsequently, Gene Ontology (GO) annotation, Kyoto Encyclopedia of Genes and Genomes (KEGG) functional interpretation, Gene Set Enrichment Analysis (https://www.gsea-msigdb.org/gsea/index.jsp), and protein-protein interaction (PPI) network analyses were conducted to analyze the consensus genes. We then conducted expressed validation of critical genes and analyses of related clinical data using UALCAN (http://ualcan.path.uab.edu/analysis.html). The pairwise correlation and prognostic role of the critical target genes were validated based on the Gene Expression Profiling Interactive Analysis 2 (GEPIA2, http://gepia2.cancer-pku.cn/#index) and their protein expression were ascertained via the Human Protein Atlas (HPA, http://www.proteinatlas.org). In addition, the diagnostic value of critical target genes was estimated using multiple-gene comparison and principal component analysis. Finally, because the tumor immune microenvironment in THCA has attracted increasing attention, we mined the Cell Type Identification By Estimating Relative Subsets Of RNA Transcripts (CIBERSORT, https://cibersort.stanford.edu) and Tumor Immune Estimation Resource (TIMER, https://cistrome.shinyapps.io/timer/) to demonstrate the relationship between miR-222-3p in THCA and immune infiltrate cells.

## Material and Methods

### Evaluation of Differential Expressisssson of miR-222-3p Expression

MiRNA expression data were downloaded from TCGA and GEO databases. The inclusion criteria for the datasets in the GEO database were as follows: 1) samples from Homo sapiens, and 2) sample size of each group ≥3. All data was normalized using the min-max normalization method. An unpaired *t*-test was performed, and the mean and standard deviations of expression levels were calculated to examine the differential miR-222-3p expression in THCA and non-cancerous tissues. Statistical significance was set at *P*_adj_ < 0.05.

### Identification of Possible Target Gene

To predict the possible target genes of miR-222-3p, we utilized the online analysis website miRWalk3.0, including four prediction algorithms, TargetScan, miRDB, mirtarbase, and TarPmiR(Sticht et al., 2018). To improve the accuracy, the calculated mRNA expressions from the TCGA database were analyzed using R software package edgeR, and the DEGs were obtained. The overlapped genes of DEGs and the possible target genes were identified as the consensus genes using a Venn diagram.

### Pathway Analysis

To elucidate the biological functions and signal transduction pathways of miR-222-3p and its target genes, GO annotation and KEGG functional interpretation were performed for the consensus genes using database for Annotation, Visualization and Integrated Discovery (DAVID) (Huang et al., 2009; Kanehisa et al., 2017; The Gene Ontology, 2019). The GSEA analysis was performed to reveal the signature pathways and biological processes(Mootha et al., 2003; Subramanian et al., 2005). Statistical significance was set at *p* < 0.05.

### Construction of PPI Network and Module Analysis

The PPI network and module analysis helped identify the critical genes of miR-222-3p and verified their interaction. We input the consensus genes into Cytoscape 3.7.2(Shannon et al., 2003), and imported the network data from the public database, Search Tool for the Retrieval of Interacting Genes (STRING), to construct the PPI network(Szklarczyk et al., 2019). Parameters of the construction of PPI network were as follows: species = Homo sapiens, confidence (score) cutoff = 0.40, and maximum additional interactors = 0. Module analysis was performed using the MCODE and cytoHubba plug-in. The MCODE plug-in identified the key module with the following truncation criteria: degree cutoff = 2, node score cutoff = 0.2, k-core = 2, max. depth = 100. The cluster network with genes with the highest MCODE score (6.0) was confirmed as the core network. The topological algorithm MCC in the cytoHubba plug-in was applied to explore important nodes in the PPI network.

### External Validation of Critical Genes

#### Differential Expression of Critical Genes and Analyses of Related Clinical Data

Based on TCGA gene expression and clinical data, UALCAN was used to verify the expression of critical genes and perform the analyses of related clinical data in samples of THCA and normal thyroid (Chandrashekar et al., 2017). The 505 THCA samples and 59 normal thyroid tissue samples were involved in validation of expression difference of critical genes and correlation analyses between critical genes and gender, age, race, tumor stage and lymph node metastasis.

#### Survival Analysis and Integrated Research

GEPIA2 could provide survival and integrated analysis, allowing researchers to select their genes and cancers of interest for evaluation of prognosis. We input the critical genes into the Survival Plots module, and the Kaplan-Meier curves were plotted with the log rank *p* value and hazard ratio (HR) with 95% confidence intervals (95% CI). Meanwhile, we conducted a multiple gene comparison and principal component analysis for the critical genes to explore their diagnostic value.

#### Correlation Analysis

To illustrate the association of miR-222-3p with the critical genes, we performed a Spearman’s correlation analysis based on 500 thyroid carcinoma samples utilizing the LinkedOmics database(Vasaikar et al., 2018). The pairwise correlation among the critical genes was also verified using the database (Sample size = 501). Statistical significance was set at *p* < 0.05.

#### Protein Expression of Critical Genes

To validate the protein expression of the critical genes in THCA and adjacent normal tissues, the immunohistochemistry results of the critical genes were acquired from the Human Protein Atlas (HPA, http://www.proteinatlas.org), and the staining intensity of the antibody revealed the protein expression of critical genes(Uhlen et al., 2015).

#### Immune Infiltration Analysis

To explore the potential relationship between the gene and immune cells in the tumor immune environment of THCA, the mRNA expression matrix from TCGA was normalized and the CIBERSORT algorithm was utilized to estimate the composition of 22 human immune cells in tumor immune environment(Newman et al., 2015). Then, the composition of immune cells in THCA and normal tissue group was compared with an unpaired *t*-test. Moreover, we used the TIMER algorithm to illustrate the correlation between the expression of critical genes and the abundance of six tumor-infiltrating immune cells (CD4 + T cells, CD8 + T cells, B cells, neutrophils, dendritic cells, and macrophages) in THCA(Li et al., 2017). The correlation values were processed using purity-corrected partial Spearman’s correlation. In general, genes upregulated in the microenvironment are negatively associated with tumor purity, whereas genes upregulated in tumor cells are positively associated with tumor purity. All *p*-values < 0.05 were considered significant.

## Results

### Differential miR-222-3p Expression

**FIGURE 1:**
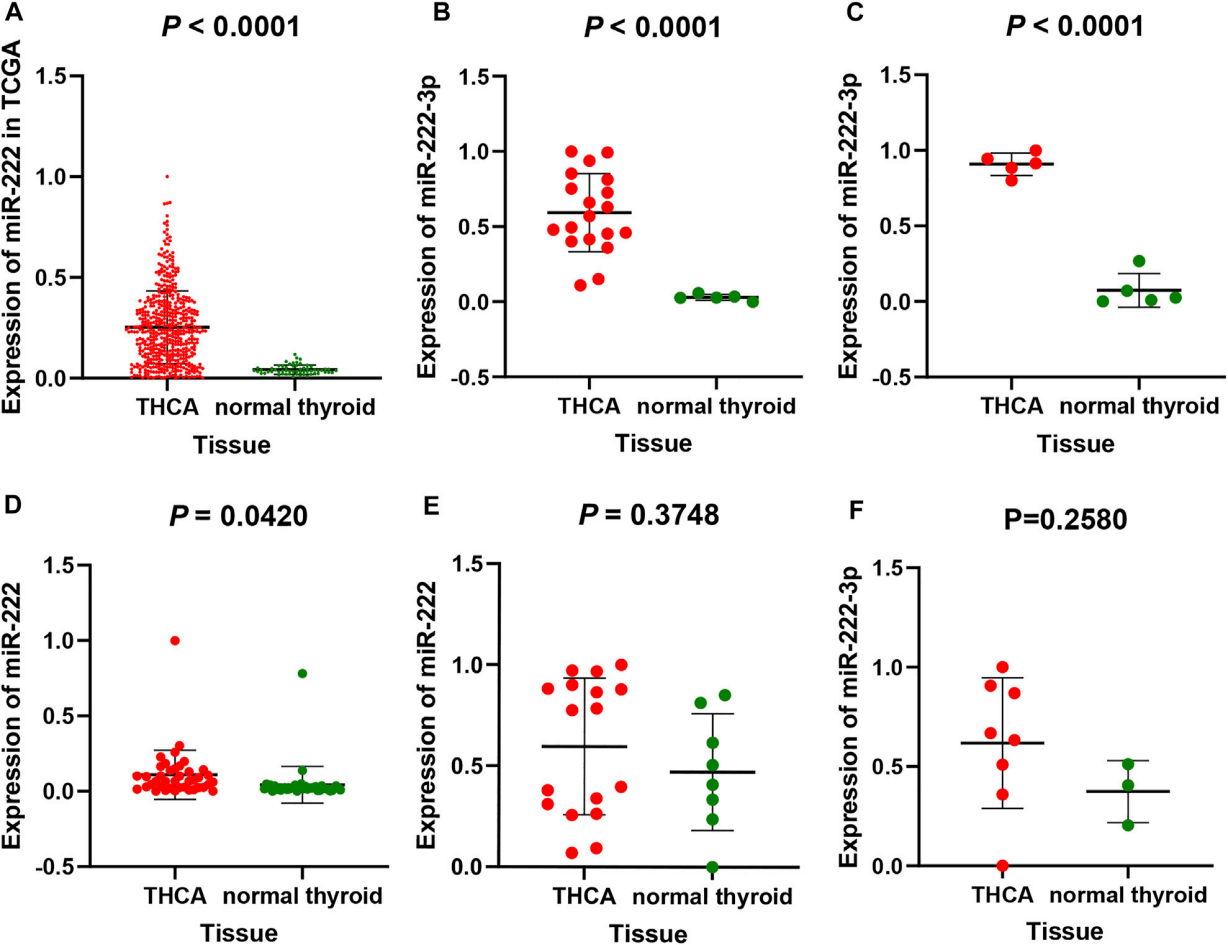
miR-222-3p differential expression in THCA and non-cancerous tissues based on TCGA database **(A)** and GEO database, including gse73182 **(B)**, gse113629 **(C)**, gse40807 **(D)**, gse62054 **(E)** and gse97070 **(F)** dataset. FA stands for thyroid follicular adenoma.

From the TCGA and GEO database, a total of 603 THCA and 120 normal thyroid tissue samples were compared in the present study. The miR-222-3p expression in THCA samples was significantly upregulated compared to that in normal thyroid tissue (*p* < 0.001). The mean ± standard deviation were 0.25250 ± 0.18090 and 0.04267 ± 0.02269, respectively (Figure 1A). As shown in Figure 1B–F, the study included five datasets from the GEO database. Among them, three databases (gse73182, gse113629, and gse40807) shared the same result, consistent with that of TCGA (*p* < 0.05), whereas no statistical significance was found in the two remaining datasets (gse62054 and gse97070).

### Possible Target Genes of miR-222-3p in THCA

**FIGURE 2:**
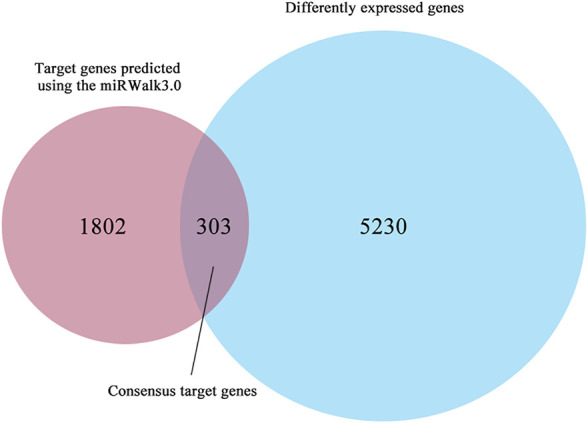
The Venn diagram shows a total of 303 consensus genes.

Possible target genes were determined using the target gene prediction platform miRWalk3.0 and differential expression based on TCGA. The differential expression analysis involved 510 THCA and 58 matched group, in which 5,533 differently expressed genes (|log2FC| > 1) were identified by edgeR. In addition, 2,105 possible target genes were predicted using the miRWalk3.0. Consequently, we regarded 303 overlapping genes as consensus target genes of miR-222-3p (Figure 2).

### Pathway Analysis

**FIGURE 3:**
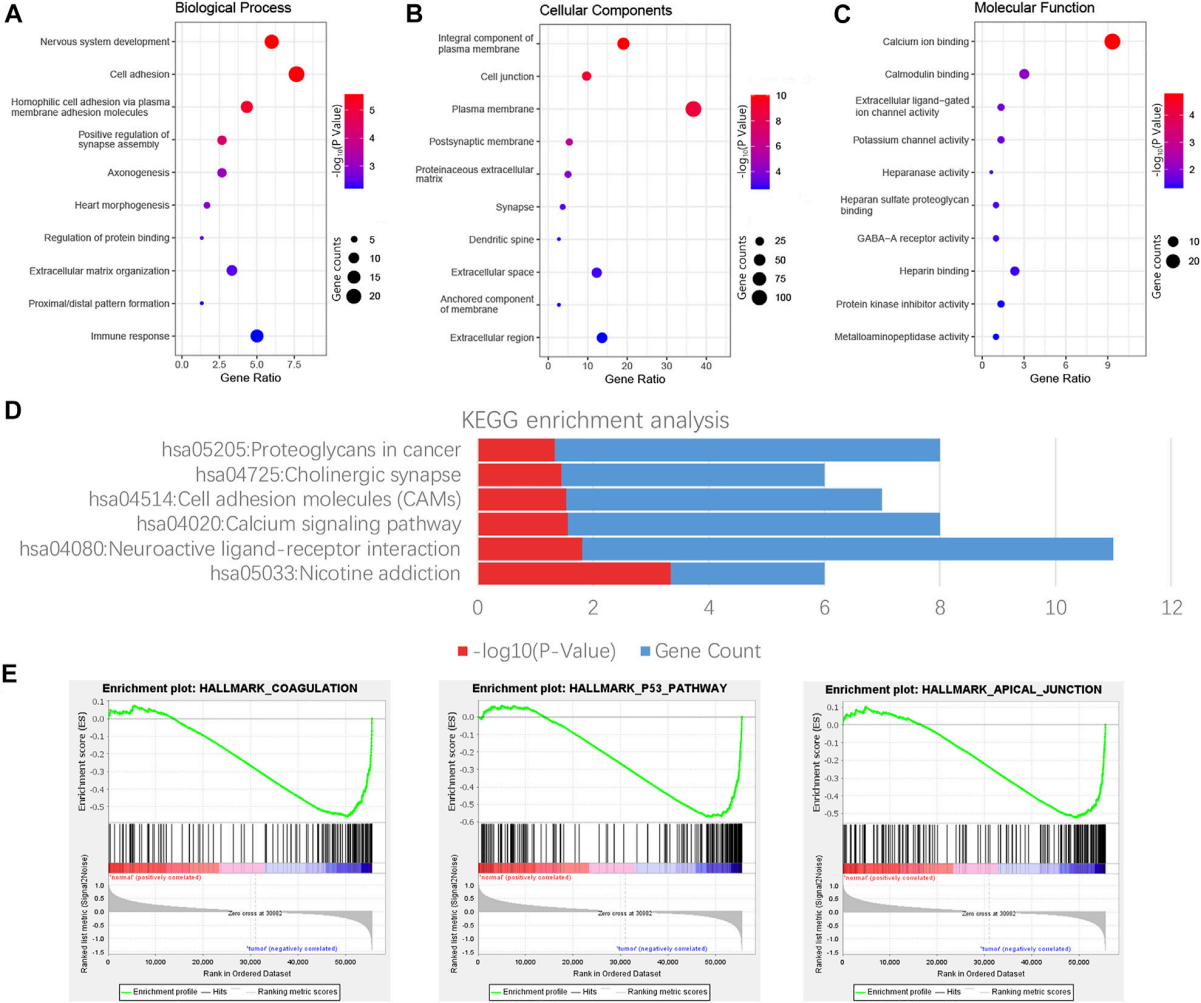
Functional enrichment analysis of consensus genes. GO enrichment analysis results include Biological process (BP), cell composition (CC), and molecular function (MF), and each part displays 10 terms **(A–C)**. The result shown by KEGG is six possible signature pathways for enrichment of consensus genes **(D)**. Pathway enrichment plots of three gene sets by performing GSEA **(E)**.

GO and KEGG enrichment analyses of 303 consensus genes were performed using DAVID. GO annotation showed that 28, 26, and 11 clustered terms were statistically significant in biological process (BP), cellular component (CC), and molecular function (MF), respectively, and a total of six significant pathways were verified by KEGG analysis. These noticeably enriched GO terms and KEGG pathways may play an important role in the mechanism of thyroid carcinoma, which might contribute to appropriate tailoring of treatment for patients. The BP GO term “nervous system development,” CC GO term “integral component of plasma membrane” and the MF GO term “calcium ion binding” had the smallest *p*-value, indicating the closest connection with the tumor (Figures 3A–C). For the KEGG pathway, nicotine addiction had the smallest *p* value, whereas neuroactive ligand-receptor interaction had the largest number of consensus genes **(** Figure 3D). The results demonstrated three significantly enriched gene sets from the following pathways: HALLMARK_COAGULATION, HALLMARK_P53_PATHWAY, and HALLMARK_APICAL_JUNCTION **(** Figure 3E).

### Identification of Critical Genes

**FIGURE 4:**
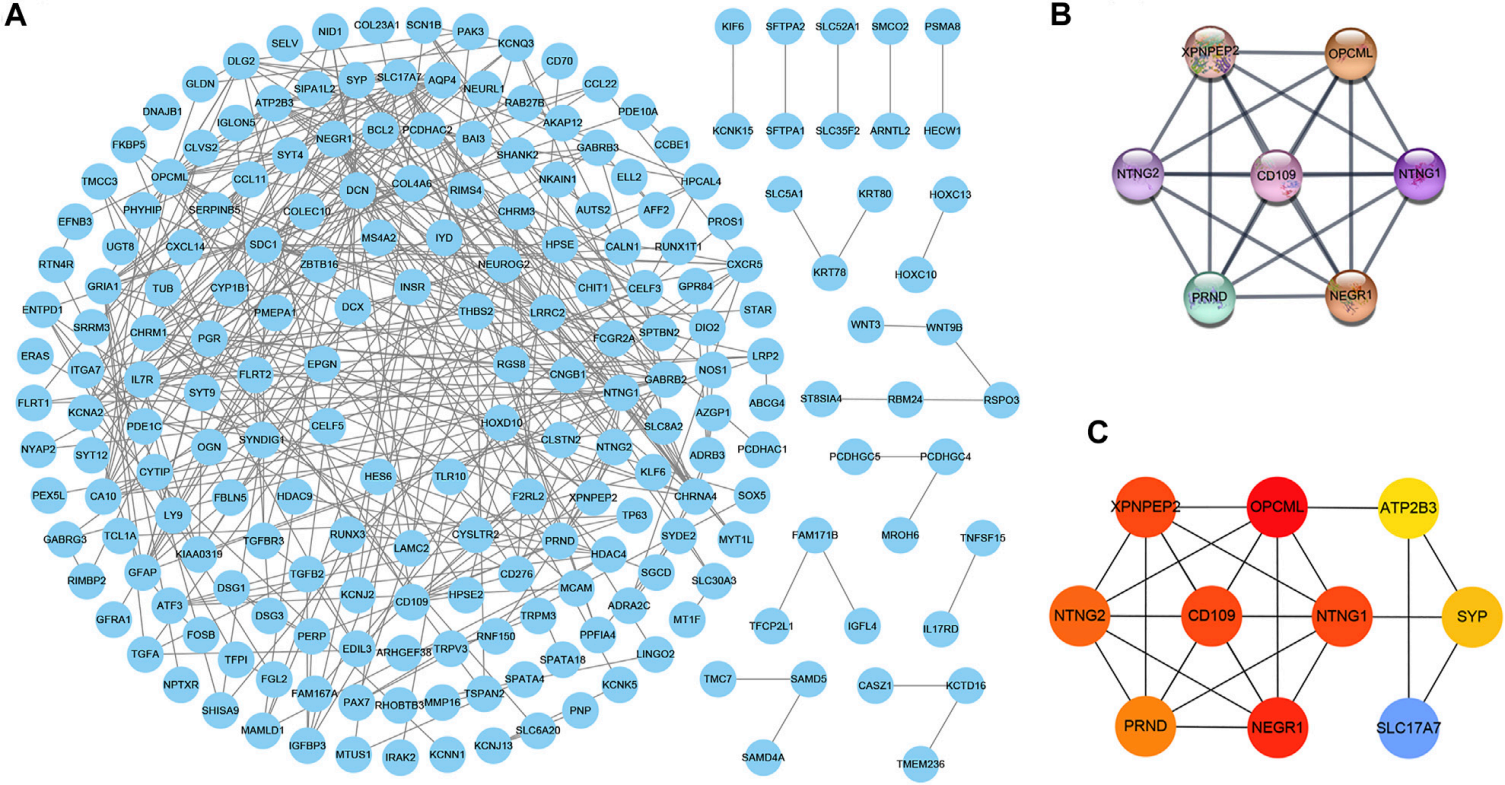
The PPI network composed of 303 consensus genes, in which each node and edge represents each gene-encoded protein and link between proteins, respectively **(A) (B)** The top scored module calculated by the MCODE plug-in of Cytoscape software **(C)** The top 10 scored genes calculated by cytoHubba plug-in of Cytoscape software, and the **red** represents the score of genes. The deeper the **red,** the higher the score.

The PPI network consisted of 302 nodes and 369 edges generated by entering 303 consensus genes into the Cytoscape 3.7.2 (Figure 4A). The module analysis of the PPI network, conducted by the MCODE and cytoHubba plug-in, uncovered two core modules (Figure 4B, C). In the two above modules, except for *ATP2B3*, *SLC17A7*, and *SYP*, the remaining seven genes (*NEGR1*, *NTNG1*, *XPNPEP2*, *NTNG2*, *CD109*, *OPCML*, and *PRND*) were identified as critical genes, indicating that they may be important in the pathogenesis of THCA.

### External Validation of Critical Genes

#### Differential Expression of Critical Genes and Analyses of Related Clinical Data

**FIGURE 5:**
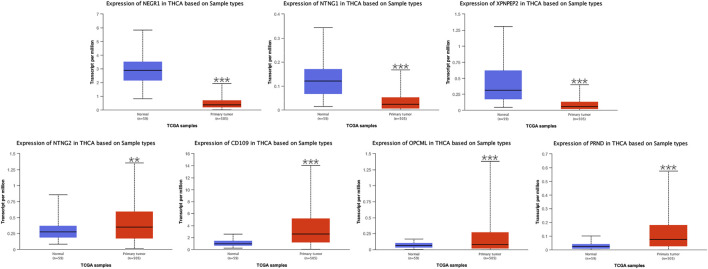
Differential expression of *NEGR1*, *NTNG1*, *XPNPEP2*, *NTNG2*, *CD109*, *OPCML*, and *PRND* based on UALCAN (^∗^ *p* < 0.05, ^∗∗^ *p* < 0.01, and ^∗∗∗^ *p* < 0.001).

Seven critical genes were identified in the module closest to the PPI network. Based on the UALCAN, we evaluated differential expression of the seven critical genes and preformed the analyses of related clinical data in samples of THCA and normal thyroid. In differential expression of critical genes (Figure 5), three of the seven genes (*NEGR1*, *NTNG1*, and *XPNPEP2*) showed low expression, and the remaining critical genes (*NTNG2*, *CD109*, *OPCML*, and *PRND*) were highly expressed in THCA samples, comparing to normal thyroid samples. In terms of gender (Supplementary Figure S1), differential expression of seven critical genes all had statistical significance in the groups of male and female. In terms of age (Supplementary Figure S2), differential expression of *NEGR1*, *CD109*, and *PRND* had statistical significance in all age groups. Differential expression of *NTNG1*, *XPNPEP2*, and *OPCML* had statistical significance in the groups of 21–40, 41–60, and 61–80 years, but not in the group of 81–100 years. And differential expression of *NTNG2* had statistical significance in the group of 21–40 years, but not in the group of 41–60, 61–80, or 81–100 years. In terms of race (Supplementary Figure S3), differential expression of *NTNG1*, *XPNPEP2*, *CD109*, *OPCML*, and *PRND* had statistical significance in all race groups. Differential expression of *NEGR1* had statistical significance in the groups of Caucasian and Asian, but not in the group of African-American. And differential expression of *NTNG2* had statistical significance in the group of Caucasian, but not in the groups of African-American or Asian. In terms of tumor stage (Supplementary Figure S4), differential expression of *NEGR1*, *NTNG1*, *XPNPEP2*, *CD109*, and *PRND* had statistical significance in all tumor stage groups. Differential expression of *OPCML* had statistical significance in the groups of stage1, 3, and 4, but not in the group of stage2. And differential expression of *NTNG2* had statistical significance in the group of stage1, but not in the groups of stage2, 3, or 4. In terms of lymph node metastasis (Supplementary Figure S5), differential expression of *NEGR1*, *NTNG1*, *XPNPEP2*, *CD109*, *OPCML*, and *PRND* had statistical significance in the groups of N0 and N1. And there was no statistical significance in differential expression of *NTNG2* in the groups of N0 or N1.

#### Prognostic Value of Critical Genes

**FIGURE 6:**
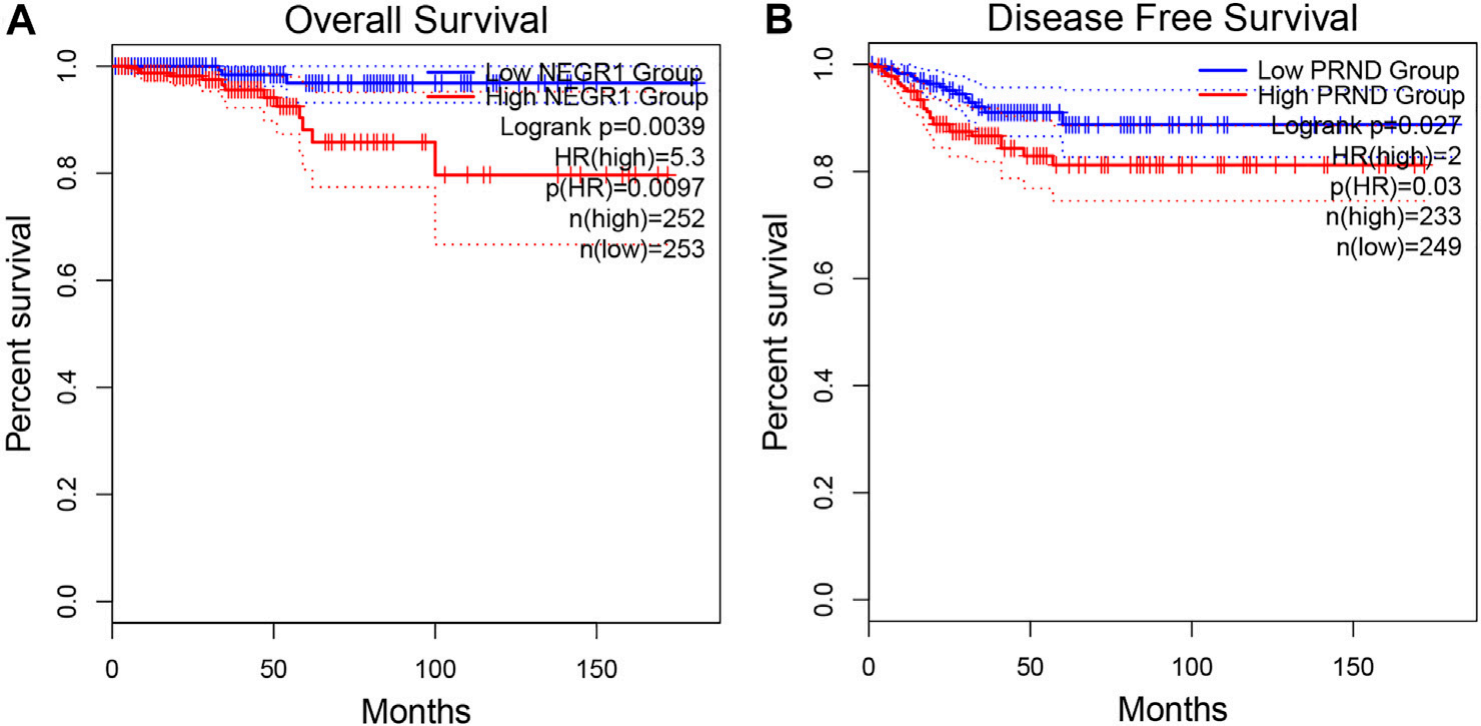
Overall survival analysis of *NEGR1* **(A)** and disease free survival analysis of *PRND* **(B)** based on GEPIA2.

A total of 505 THCA patients underwent survival analysis using the Kaplan-Meier (KM) curve. Interestingly, high *NEGR1* expression was significantly correlated with worse OS (HR = 5.3, *p* = 0.0097), whereas DFS was not significantly different (Figure 6A). On the contrary, low *PRND* expression was significantly correlated with better DFS (HR = 2, *p* = 0.03), whereas the OS results were not significantly different (Figure 6B). For the other critical genes, results of the survival analysis were not statistically significant.

#### Diagnostic Value of Seven-Gene Biomarker

**FIGURE 7:**
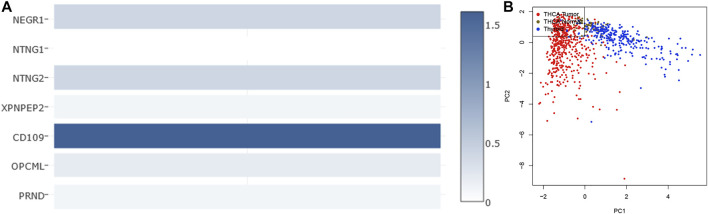
Multiple-gene comparison analysis **(A)** and principal component analysis **(B)** of the seven-gene biomarker in THCA.

The two analyzed modules on GEPIA2, multiple-gene comparison and principal component analysis, were utilized to estimate the diagnostic value of the seven-gene biomarker. Among them, *CD109* exhibited the highest expression level in THCA samples via multiple-gene comparison, followed by *NTNG2*, *NEGR1*, *OPCML*, *XPNPEP2*, *PRND*, and *NTNG1*(Figure 7A). Notably, results of principal component analysis with TCGA tumor data, TCGA normal data, and GTEx thyroid data revealed that the seven-gene biomarker could contribute to effectively differentiate THCA from normal thyroid samples (Figure 7B), suggesting its potential value in the diagnosis of THCA.

#### Correlation Analysis

**FIGURE 8:**
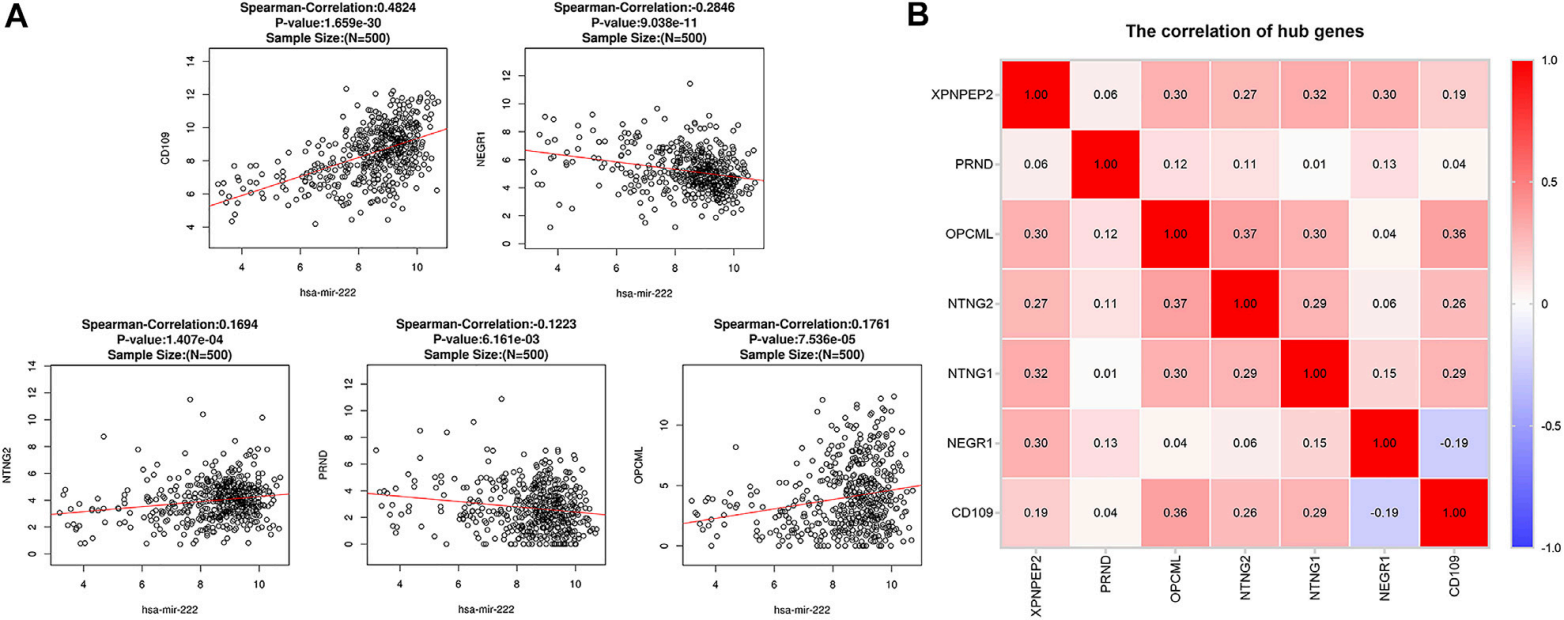
Correlation analysis between miR-222-3p and *CD109*, *NTNG2*, *OPCML*, *NEGR1* and *PRND* in THCA **(A)**. Pairwise correlation analysis of the expression of critical genes in THCA **(B)**. **Red:** positive correlation; **blue:** negative correlation.

Spearman’s correlation analysis revealed that five of the seven critical genes were significantly correlated with miR-222-3p expression. As shown in Figure 8A, *CD109* (r = 0.482, *p* < 0.001), *NTNG2* (r = 0.169, *p* < 0.001), and *OPCML* (r = 0.176, *p* < 0.001) were positively correlated with miR-222-3p, whereas *NEGR1* (r = −0.285, *p* < 0.001) and *PRND* (r = −0.122, *p* < 0.01) were negatively correlated. However, the correlation of *NTNG1* (*p* = 0.29) and *XPNPEP2* (*p* = 0.15) with miR-222-3p was not statistically significant. Interestingly, a negative correlation (cor = -0.19) only existed between *NEGR1* and *CD109*, while the other groups exhibited positive correlations (Figure 8B).

#### Protein Expression of Critical Genes

**FIGURE 9:**
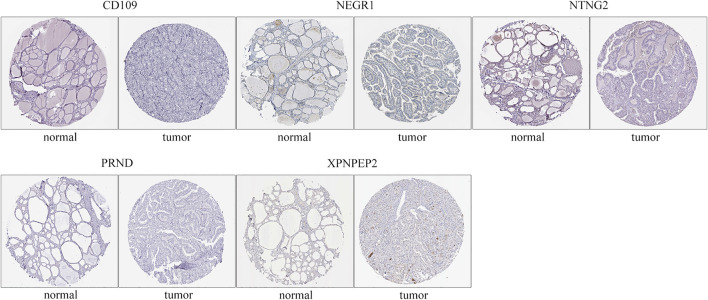
Protein expression of critical genes. Images were obtained from the HPA database.

The immunohistochemistry results of critical genes of THCA in the HPA database were investigated to ascertain their protein expression. It is worth noting that only *NEGR1* had a marked negative protein expression in carcinoma tissues compared to normal gland tissues, consistent with the above differential expression of critical genes (Figure 9), whereas *CD109*, *NTNG2*, *PRND*, and *XPNPEP1* showed no difference in protein expression between normal and tumor tissues. The immunohistochemistry results of *NTNG1* and *OPCML* were not available for HPA.

#### Immune Infiltration Analysis

**FIGURE 10:**
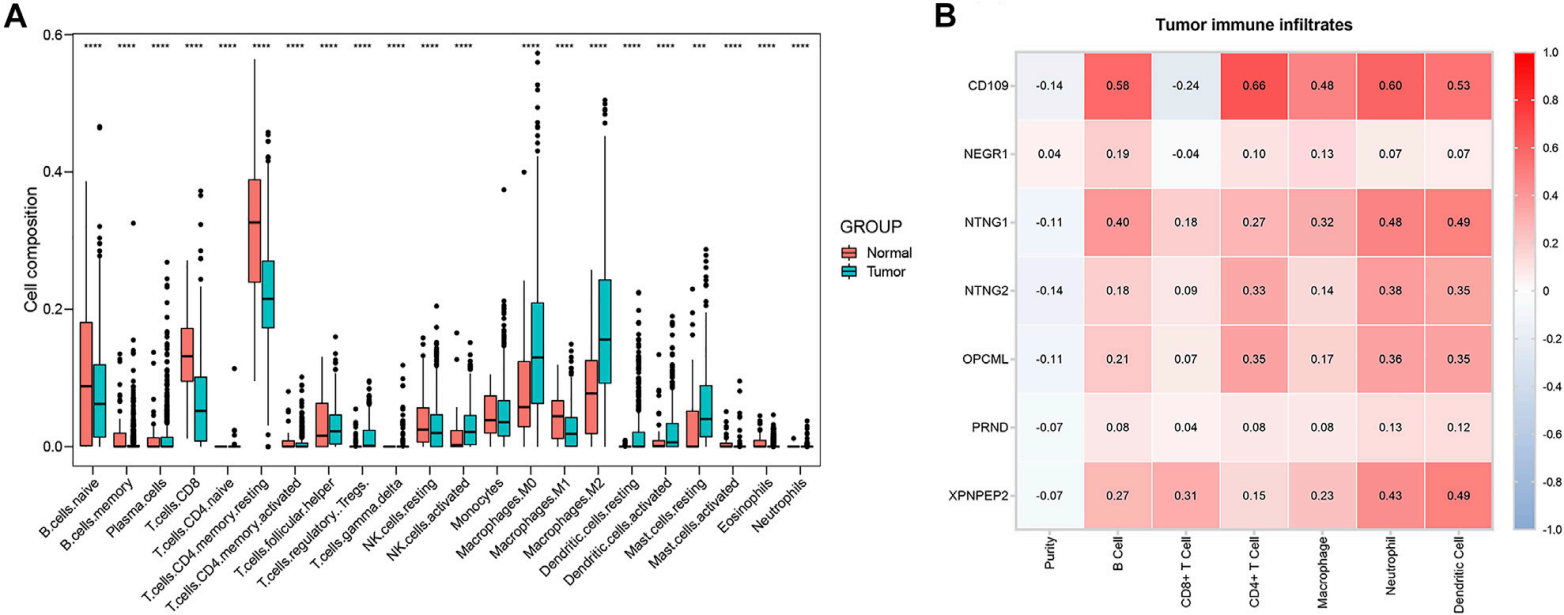
Boxplot of 22 immune cells composition in THCA and normal tissue group **(A)**. The correlation of critical genes and tumor immune infiltrates analyzed via the Tumor Immune Estimation Resource (TIMER) platform **(B)**. **Red:** positive correlation; **blue:** negative correlation.

The composition of 22 immune cells in tumor immune environment was estimated. In TCGA databases, the THCA and normal tissue group showed a significant difference for the composition of 21 immune cells (naive B cells, memory B cells, plasma cells, CD8^+^ T cells, naive CD4^+^ T cells, memory-resting CD4^+^ T cells, memory-activated CD4^+^ T cells, follicular helper T cells, regulatory T cells (Tregs), gamma delta T cells, resting NK cells, activated NK cells, M0 macrophages, M1 macrophages, M2 macrophages, resting dendritic cells, activated dendritic cells, resting mast cells, activated mast cells, eosinophils, neutrophils) (Figure 10A). Contributing to providing insight for immunotherapeutic treatment in THCA, the tumor immune infiltrates of THCA, including B cells, CD4^+^ T cells, CD8^+^ T cells, neutrophils, macrophages and dendritic cells, were analyzed via the TIMER platform. *CD109* demonstrated the highest positive correlation with B cells (cor = 0.58, *p* < 0.001), CD4^+^ T cells (cor = 0.66, *p* < 0.001), macrophages (cor = 0.48, *p* < 0.001), neutrophils (cor = 0.60, *p* < 0.001), and dendritic cells (cor = 0.53, *p* < 0.001), and the highest negative correlation with CD8^+^ T cells (cor = −0.24, *p* < 0.001) (Figure 10B). Moreover, *NTNG1* and *XPNPEP2* were also closely related to several types of immune-infiltrate cells (Figure 10B). In addition, an insignificant correlation was found between all types of immune-infiltrate cells and *NEGR1* and *PRND* (Figure 10B).

## Discussion

Based on the TCGA and GEO databases, we confirmed miR-222-3p overexpression in THCA, consistent with previous studies(Paskas et al., 2015; Borrelli et al., 2017). Moreover, elevated miR-222-3p expression was associated with high-risk characteristics, including tumor invasion, lymph node metastasis, and recurrence that may be closely related to the poor prognosis of THCA patients (Aragon Han et al., 2015; Lima et al., 2017; Gomez-Perez et al., 2019). Visone et al. reported that miR-221 and miR-222 regulate the cell cycle by targeting p27^Kip1^ in human thyroid papillary carcinomas, while abnormal cell cycle leads to malignant transformation. Moreover, Huang et al. indicated that miR-222 promotes tumor invasion and metastasis in papillary thyroid cancer by targeting *PPP2R2A*(Huang et al., 2018). However, few researchers have focused on the prognostic and diagnostic value of miR-222-3p and its target genes and the correlation between target genes and immune invasion.

In the present study, we aimed to elucidate the biological functions of miR-222-3p and its target genes in THCA. Differential expression analysis of miR-222-3p based on the GEO and TCGA database indicated the upregulation of miR-222-3p expression in THCA; 303 consensus genes were obtained by overlapping the target genes predicted by miRWalk3.0 and DEGs from the TCGA database. To search for enriched pathways of miR-222-3p and its target genes, we acquired three possible signature pathways using the GSEA analysis. Moreover, using the STRING public platform, the PPI network was constructed, and seven genes (*NEGR1*, *NTNG1*, *XPNPEP2*, *NTNG2*, *CD109*, *OPCML*, and *PRND*) were identified as critical genes through module analysis using the Cytoscape software.

We discovered close relationships between critical genes and prognosis of THCA. Results confirmed that *NEGR1* correlated with the overall survival (OS) of THCA, while *PRND* was associated with disease-free survival (DFS) and THCA. Neuronal growth regulator 1 (*NEGR1*) is a member of the immunoglobulin LON (IgLON) family, which pertains to GPI-anchored cell adhesion molecules (CAMs), consistent with the pathways of KEGG enrichment analysis(Kubick et al., 2018). Previous studies revealed that *NEGR1* is commonly downregulated as a tumor suppressor gene in many cancers, including neuroblastoma, breast, colon, and kidney cancers(Takita et al., 2011; Hiemer et al., 2014; Huang et al., 2020). By investigating the genetic landscape of anaplastic thyroid cancer, Woodward et al. confirmed that the *NEGR1* frequent deletion were associated with the tumorigenesis of anaplastic thyroid carcinoma(Woodward et al., 2017). This study showed that *NEGR1* was related to the overall survival of THCA patients, and a low *NEGR1* expression may drive tumor progression by abnormal cell adhesion. However, further studies are needed to verify this hypothesis. Prion-like protein doppel (*PRND*), a paralog of the prion (PrP) protein, was upregulated in many malignant diseases, including astrocytomas, osteosarcoma, acute myeloid leukemia, and myelodysplastic syndromes(Travaglino et al., 2005; Comincini et al., 2007; Sollazzo et al., 2012; Al-Hilal et al., 2016). To date, no study has elucidated the regulatory mechanism of *PRND* in thyroid cancer. In our study, *PRND* had a high level expression in THCA, indicating poor DFS. Nevertheless, more research is needed to explore the target signature pathway.

Even though *NEGR1* and *PRND* exhibited prognostic significance in THCA, their pairwise correlations indicated that the remaining critical genes, *CD109*, *NTNG1*, *NTNG2*, *XPNPEP2*, and *OPCML*, also play important roles in the regulation of biological processes. The analyses of related clinical data revealed that critical genes were all significantly related to tumor stage and lymph node metastasis of THCA. In addition, multiple-gene comparison and principal component analysis of critical genes revealed that they had potential diagnostic value in THCA, which indicated that their conjoint analysis offered a method for effectively distinguishing THCA from normal thyroid tissue. Therefore, seven critical genes were suggested as biomarkers for the precise diagnosis of THCA. These results provide insights into the role of multiple genes and interactive networks in the multilevel regulation of the molecular mechanism of THCA.

Previous studies revealed that immune cells in the tumor microenvironment play an essential role in the occurrence and development of THCA(Zhuang et al., 2020). As an important part of the complex tumor microenvironment, immune infiltrate cells are correlated with the biological processes of cancers and the survival of cancer patients. In this study, the analysis of tumor immune infiltrates revealed that gene signature of THCA had a close relationship with 21 types of immune cells in tumor immune environment. And *CD109* had the highest positive correlation with B cells, CD4^+^ T cells, macrophages, neutrophils, and dendritic cells, and the highest negative correlation with CD8^+^ T cells. *NTNG1* and *XPNPEP2* also exhibited a high correlation with a variety of immune cells. Overall, the results of immune infiltrates illustrated that miR-222-3p was pertinent to the regulation of the immune microenvironment, mainly through targeting *CD109*, *NTNG1*, and *XPNPEP2* in THCA.

Inevitably, there were certain limitations to our study. As the datasets were obtained from different databases and platforms, an uncertain systematic bias might exist. Furthermore, although enriched analysis was performed to preliminarily investigate the regulatory function of miR-222-3p and its target genes in THCA, the particular mechanism between miR-222-3p and critical genes requires further *in vitro* and *in vivo* experiments. Nevertheless, the results of this study are significant as they highlight on promising biomarkers of prognosis, diagnosis, and immune infiltrates.

## Conclusion

In summary, using integrated bioinformatics analysis, we demonstrated that miR-222-3p was upregulated in THCA. The elevated miR-222-3p expression may result in downregulated *NEGR1* expression and elevated *PRND* expression. Downregulated *NEGR1* expression indicating a positive prognosis of THCA, whereas elevated *PRND* expression indicated poor prognosis. *CD109* may be capable of regulating B cells, CD4+T cells, macrophages, neutrophils, and dendritic cells in immune microenvironment of THCA. Hence, the critical target genes of miR-222-3p, which indicate potential prognostic value, play an essential role in the infiltration of immune cells and could act as potential targets for the treatment of THCA patients.

## Data Availability

Publicly available datasets were analyzed in this study. This data can be found here: The Cancer Genome Atlas (https://portal.gdc.cancer.gov/repository).
