# Supplementary material for: Prognostic and Immune-Infiltrate Significance of miR-222-3p and Its Target Genes in Thyroid Cancer
Source: Front Genet. 2021 Oct 19;12:710412. doi: 10.3389/fgene.2021.710412 (PMC8562566; doi:10.3389/fgene.2021.710412)
Supplement: Supplementary file 1 [file DataSheet1.docx]

**Prognostic and immune-infiltrate significance of miR-222-3p and its target genes in thyroid cancer**

**Figure S1**. In terms of gender, differential expression of NEGR1, NTNG1, XPNPEP2, NTNG2, CD109, OPCML, and PRND based on UALCAN (* P<0.05, ** P<0.01, *** P<0.001, - P>0.05).

**Figure S2**. In terms of age, differential expression of NEGR1, NTNG1, XPNPEP2, NTNG2, CD109, OPCML, and PRND based on UALCAN (* P<0.05, ** P<0.01, *** P<0.001, - P>0.05).

**Figure S3**. In terms of race, differential expression of NEGR1, NTNG1, XPNPEP2, NTNG2, CD109, OPCML, and PRND based on UALCAN (* P<0.05, ** P<0.01, *** P<0.001, - P>0.05)

**Figure S4**. In terms of tumor stage, differential expression of NEGR1, NTNG1, XPNPEP2, NTNG2, CD109, OPCML, and PRND based on UALCAN (* P<0.05, ** P<0.01, *** P<0.001, - P>0.05).

**Figure S5**. In terms of lymph node metastasis, differential expression of NEGR1, NTNG1, XPNPEP2, NTNG2, CD109, OPCML, and PRND based on UALCAN (* P<0.05, ** P<0.01, *** P<0.001, - P>0.05).


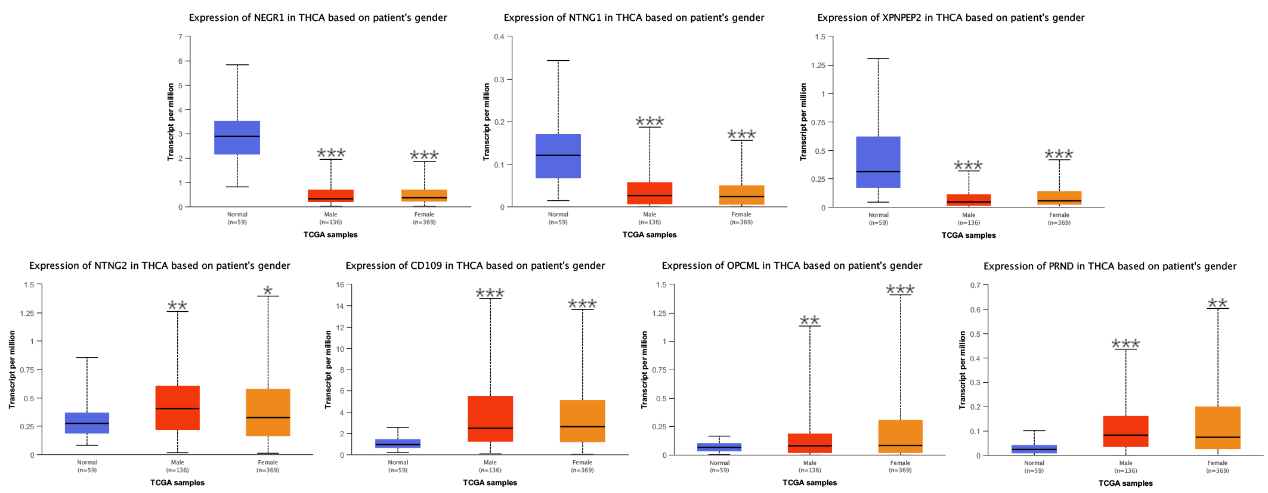


Figure S1


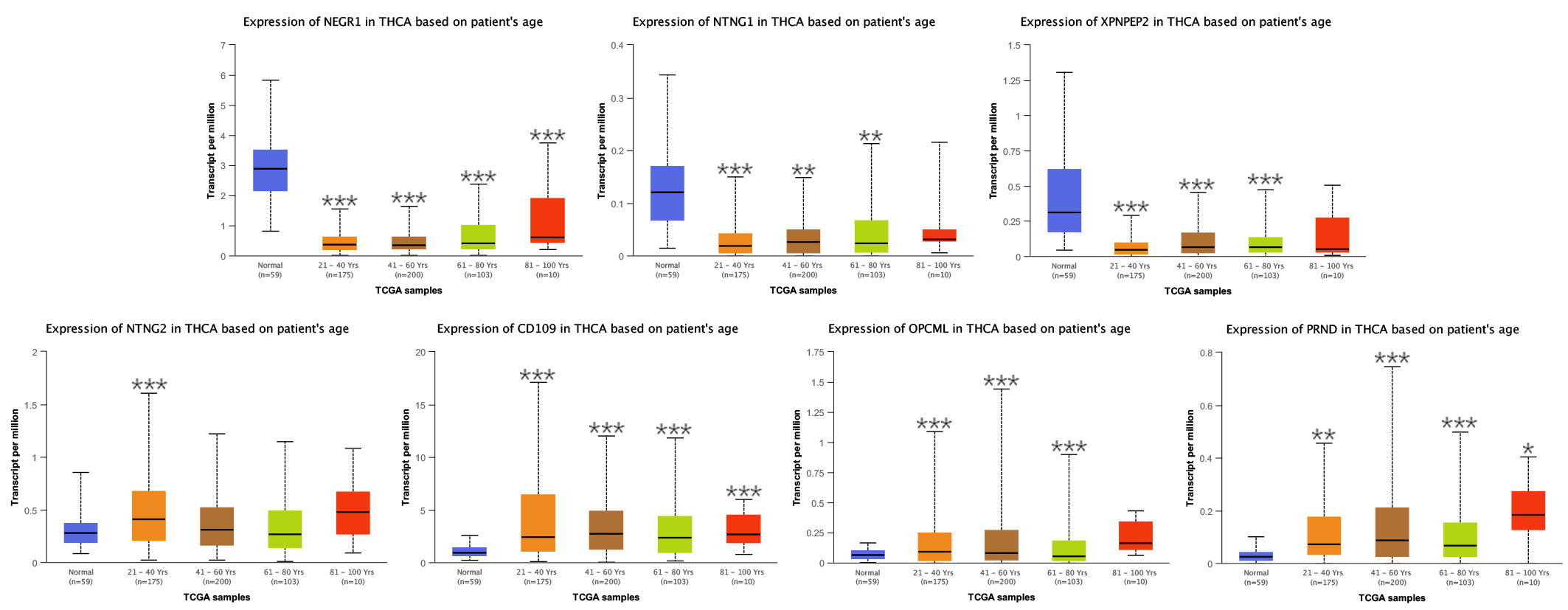


Figure S2


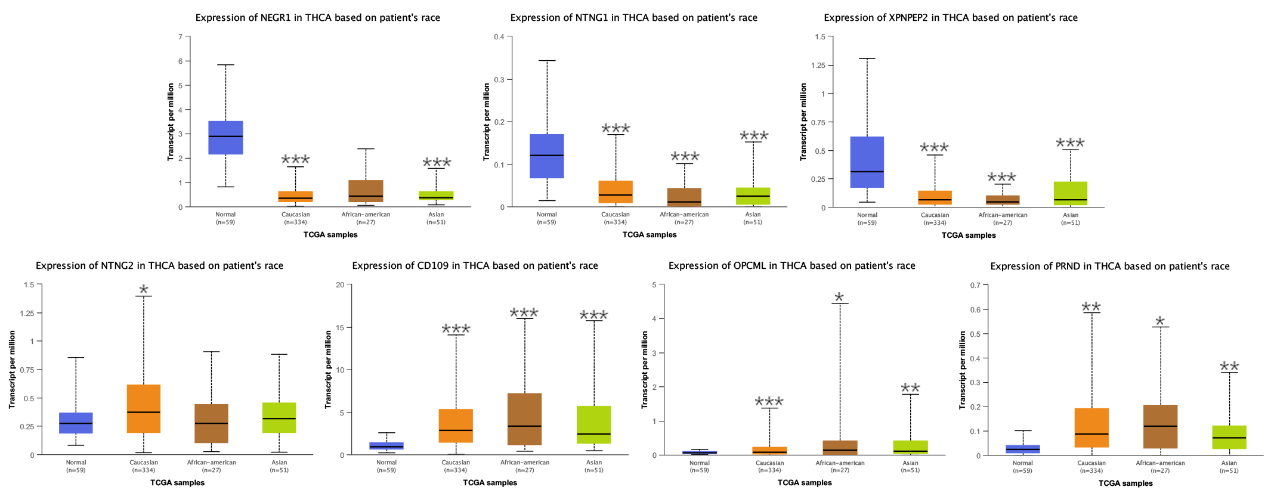


Figure S3


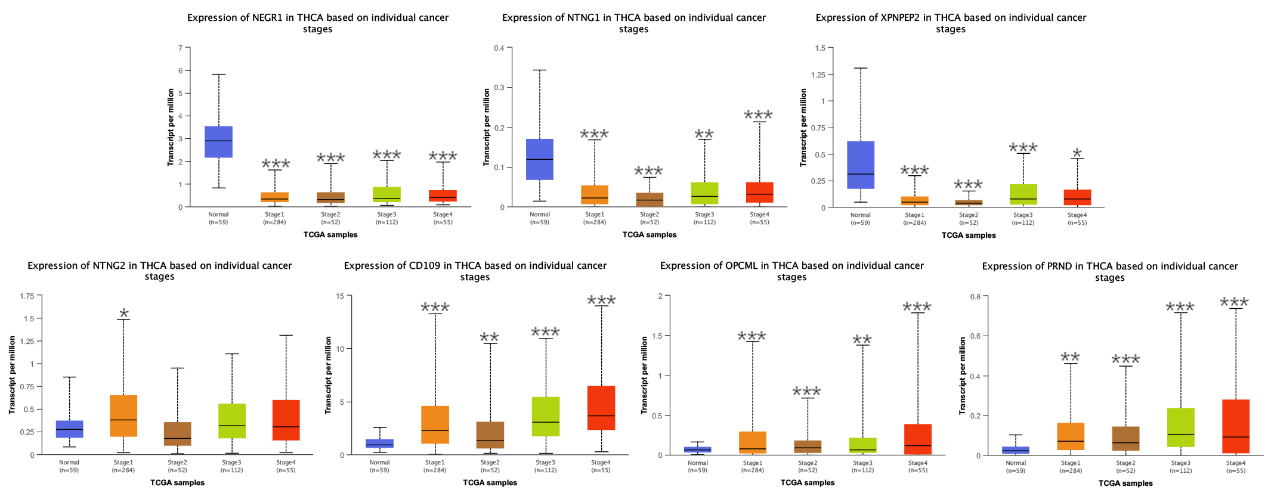


Figure S4


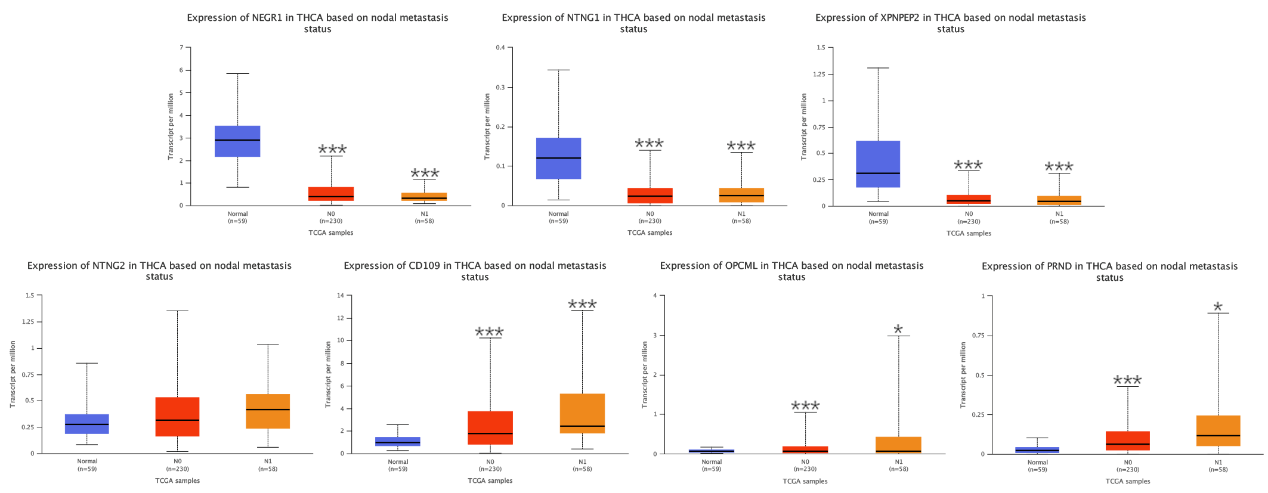


Figure S5
